# Proteomics Approaches for Discovering Novel Protein Biomarkers in Inflammatory Bowel Disease-Related Cancer

**DOI:** 10.3390/biom15091328

**Published:** 2025-09-17

**Authors:** Tommaso Saccon, Matilde Bergamo, Cinzia Franchin

**Affiliations:** 1 Department of Pharmaceutical and Pharmacological Sciences, University of Padova, Via F. Marzolo 5, 35131 Padova, Italy; tommaso.saccon@studenti.unipd.it (T.S.); matilde.bergamo96@gmail.com (M.B.); 2 Department of Pharmaceutical and Pharmacological Sciences, Mass Spectrometry Facility (MS@DSF), University of Padova, Via F. Marzolo 5, 35131 Padova, Italy

**Keywords:** IBD, proteomics, IBD-related cancer, gut inflammation, IBD therapies

## Abstract

Inflammatory bowel disease (IBD) is an autoimmune condition with an increasing incidence worldwide, which manifests in two pathological forms: Crohn’s disease (CD) or ulcerative colitis (UC). Both cause chronic inflammation of the digestive tract, although they can present different locations and with different symptoms. To date, the pathogenesis of IBD remains unclear. One of the major complications of these diseases is colorectal cancer. Several studies have reported a correlation between chronic intestinal inflammation and an increased risk of malignancy. Persistent inflammation damages the intestinal mucosa and epithelial wall, altering gut permeability and the local microenvironment. Moreover, the heightened activity of the immune system leads to an increased production of reactive oxygen and nitrogen species (ROS and RNS), increasing the risk of DNA mutation and cell transformation. In addition, some current therapies used to treat IBD and induce remission may contribute to carcinogenesis or impair immune surveillance due to their immunosuppressive activity. The management of cancer risk for IBD patients remains a challenge, and existing screening methods are often invasive (endoscopies, biopsies), resulting in low patient compliance. To address this unmet clinical need, researchers have started using proteomics to identify novel biomarkers that could predict cancer risk in IBD patients in a non-invasive manner. This review aims to examine the current state of knowledge regarding the correlation between IBD and cancer, with a special focus on the biomarkers discovered through proteomic approaches, and their potential application in routine clinical screening. In our view, proteomics represents a powerful and rapidly evolving strategy for biomarker discovery, with the potential to complement or even replace invasive procedures. Its future clinical impact will rely on translating current research advances into robust and accessible diagnostic tools.

## 1. Introduction

### 1.1. Inflammatory Bowel Disease

Inflammatory bowel disease (IBD) is an autoimmune condition that causes life-long chronic inflammation of the gastrointestinal tract due to genetic traits that lead to an exaggerated immune response against intestinal commensal microbial antigens. Although it has been established that the immune system plays a key role in the pathogenesis of IBD, the etiology of this disease is still not clear.

This pathological condition can manifest itself in two different ways: Crohn’s disease (CD) and ulcerative colitis (UC). These diseases are distinguished based on their location and on their effects on the intestinal wall and mucosa. UC leads to a chronically inflamed colonic mucosa and is divided into four subgroups, based on the area where it is detected: proctitis (rectum), proctosigmoiditis (sigmoid colon), distal UC (beyond sigmoid), and pancolitis (entire colon up to the cecum). CD can cause the ulceration of the wall in any part of the gastrointestinal tract (from the mouth to the anus), but it preferentially affects the ileum and the colon and can be classified in two phenotypes: perforating and stricturing.

When it is not possible to identify the type of IBD, the disease is referred as unclassified IBD (IBD-U). However, the term indeterminate colitis may be used if the disease is limited to the colonic trait of the intestine.

#### 1.1.1. Symptoms

Since IBD does not encompass one specific region of the gastrointestinal (GI) tract, its symptoms are dependent on the damaged intestinal area. Generally, due to the chronicity of this condition, the symptoms can be mild, moderate, or severe during relapses, but might be completely absent during remissions.

In addition to general symptoms (fever, weight loss, fatigue, etc.), more specific symptoms of IBD include diarrhea (associated with mucus or blood in the stool), constipation, painful bowel movements, abdominal pain, bowel movement urgency, tenesmus, nausea, vomiting [1].

#### 1.1.2. Intraintestinal and Extraintestinal Complications

Intestinal complications of IBD include bleeding in ulcerative colitis and abscesses, fistulas, perianal disease, perforation, and strictures in Crohn’s disease, with malignancy possible in both. Extraintestinal complications, mainly due to IBD therapies but sometimes chronic inflammation, encompass arthropathies, hepatic steatosis, and renal and skeletal disorders.

#### 1.1.3. Diagnosis

The diagnosis of IBD relies on a combination of clinical assessment and laboratory investigations. Clinical examination evaluates the patient’s general condition by assessing parameters such as blood pressure, temperature, pallor, etc. Laboratory analyses include both stool and blood tests. Among fecal markers, lactoferrin and calprotectin, most commonly quantified by ELISA, represent the reference indicators of intestinal inflammatory activity. Blood analyses comprise the detection of microbial agents by ELISA, the measurement of C-reactive protein through immunoturbidimetric assays, and the evaluation of erythrocyte sedimentation rate. The most common laboratory tests are shown in Table 1 and Table 2.

Another diagnostic method involves endoscopy. Although it is the most invasive of all the previously mentioned methods, it represents the gold standard for obtaining precise diagnosis, since it allows a visual assessment of the state of the intestine. In fact, endoscopy is essential to validate the presence of ulcers, lesions, fistulas, abscesses, and other IBD-related manifestations. Moreover, endoscopy facilitates the detection of early dysplastic changes that may precede malignant transformation. Importantly, it also provides the opportunity to obtain biopsy specimens, which can be further analyzed through advanced proteomic techniques to better understand disease mechanisms and discover new putative biomarkers [1].

### 1.2. Epidemiology

A systematic review published in 2012 gathered data from many articles concerning the epidemiology of IBD [2]. In this review, the authors explained how the incidence of this condition is higher in developed countries, compared to Low- and Middle-Income Countries (LMIC): this can be referred to as an “East–West gradient”. Specifically, Canada and Northern Europe were the most affected areas, followed by Australia, as shown in Table 3. Moreover, they demonstrated that IBD affects both sexes with the same incnce.

In addition, the same systematic review reported an increasing incidence of IBD worldwide by examining numerous studies and extracting relevant data. In 75% of the studies, the incidence of Crohn’s disease was found to be rising, and a similar trend was observed for ulcerative colitis in 60% of them. Moreover, the increasing incidence of IBD has also been reported in other scientific works [3,4].

Data reported in Table 3 is the latest update of an official global organization. Nevertheless, the epidemiological landscape of inflammatory bowel disease (IBD) has shifted considerably in recent decades. According to the World Gastroenterology Organization Global Guidelines (2015) [1], IBD was historically more prevalent in North America and Europe. Updated data from the Global Burden of Disease 2019, reported by Park et al. (2023) [5], demonstrate a 47% increase in global crude prevalence between 1990 and 2019, accompanied by a 19% decline in the age-standardized prevalence rate, reflecting the influence of demographic dynamics. While the overall burden remains greatest in high-income regions, the most rapid increases are now observed in East Asia and the Asia-Pacific, regions previously considered low prevalence. Moreover, marked geographical variation persists, with higher prevalence consistently documented at northern latitudes across Asia, Europe, and North America. These findings indicate a transition of IBD from a predominantly Western disorder to a condition of global relevance, underscoring the necessity of integrating historical and contemporary data to inform healthcare policy and resource allocation.

### 1.3. Pathogenesis of IBD

As already mentioned, the etiology of IBD is not yet fully understood. However, there are four main factors that seem to play a key role in the occurrence of this autoimmune condition: genetics, environment, microbial factors and immune response [6].

Regarding the genetic implications of IBD, some SNPs related to IBD were identified through some genome-wide association studies (GWAS). In addition, several genes were found to be associated with CD, UC or both. These genes are implicated in circulating levels of T-cells or of specific subsets of these cells. For instance, the Signal Transducer and Activator of Transcription 3 (STAT3) genetic locus has attracted attention, since it is essential for the differentiation of T helper 17 cells which are, among other elements, responsible for the pathological immune response in IBD [7].

Kaplan and colleagues used the UK THIN database to examine the link between environmental factors and IBD. For Crohn’s disease (CD), current and former smokers and individuals with prior appendectomy had increased risk, while air pollution was generally not associated, with the exception that patients under 23 in high–nitrogen dioxide regions were more frequently diagnosed. For ulcerative colitis (UC), ex-smokers showed higher risk, whereas appendectomy and air pollution were not significant factors; however, patients under 25 with UC were more often from areas with elevated levels of sulfur dioxide [8].

The role of the gut microbiome in the induction of chronic inflammation has drawn significant attention over the last few decades. Since the beginning of the 21st century, the involvement of Adherent-Invasive *Escherichia coli* (AIEC) strains has been demonstrated. Indeed, a 2004 study revealed the presence of invasive *E. coli* in the ileal mucosa of CD patients. However, it was understood that this could not be the only inflammation-inducing factor, since some intrusive bacteria were also associated with the mucosa of patients in state of remission (but much less in healthy controls). Moreover, this study did not find AIEC in the gut mucosa of UC patients [9]. More recently, a better understanding of the important role of the microbiome and the advancement in molecular techniques has led researchers to profile not only the intestinal flora, but its variation in pathological conditions as well. Putignani and colleagues found an overall less rich fecal microbiota in IBD patients compared to controls: an increment in terms of Actinobacteria, Fusobacteria and Proteobacteria (like AIEC) was found [10].

As to the immunological factors leading to IBD, a subset of T helper cells, Th17, has been studied due to its significant involvement in autoimmunity in mice models. It is now known that these cells promote inflammation by producing the proinflammatory cytokine IL-17. Essentially, naïve CD4^+^ T differentiation to Th17 is a cytokine-induced event that requires three cytokines: IL-6, IL-21, IL-23. IL-6 induces the expression of IL-21 in naïve CD4^+^ T cells, which has an autocrine effect, leading to IL-23 expression. IL-6 and IL-21 promote the transcription of proinflammatory IL-17 through a transcription factor, STAT3. In addition, IL-21 and IL-23 activate another protein, RORγt (an orphan nuclear receptor), whose activity promotes IL-17 expression as well [11]. Precisely, IL-17 is not a single cytokine, it is a group of cytokines classified with letters from A to F (e.g.,: IL-17A, IL-17B, etc.). IL-17A is the cytokine whose correlation with gut inflammation is the clearest: this protein induces some inflammation related pathways, such as NFκB, MAPKs and C/EBPs, which lead to the synthesis of anti-microbial peptides and other inflammation-activating molecules like cytokines and chemokines [12].

### 1.4. Treatment of IBD

Because the pathogenesis of IBD is not fully understood, drugs for this class of diseases aim more to induce remission and relieve symptoms (rectal bleeding, bloody diarrhea, abdominal cramps, pain) rather than to repair the damaged mucosa or restore normal intestinal microbiota. However, in the last few years, new drugs have been developed: these pharmaceutical products work as regulators of the immune response, so they might also be useful for long term remission, in addition to symptom relief [13].

**Figure 1:**
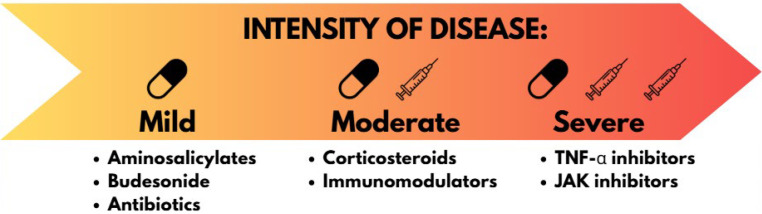
Therapeutic escalation strategy for inflammatory bowel disease, ranging from mild to severe cases.

As illustrated in Figure 1, the choice of treatment depends on the intensity of symptoms (referred as mild-to-moderate IBD or moderate-to-severe IBD).

According to the European Crohn’s and Colitis Organization (ECCO) guidelines issued in 2017 and 2022, almost all of the pharmaceutical products available for UC patients can be used both for induction and for maintenance of remission, the only exceptions are budesonide and systemic corticosteroids, which are not used for maintenance of remission, and thiopurines, which are not recommended for inducing it. For mild-to-moderate UC, the recommended drugs are 5-ASA, budesonide and thiopurines, whereas for moderate-to-severe UC systemic corticosteroids, infliximab (monotherapy), adalimumab (monotherapy), ustekinumab, vedolizumab are recommended.

In the 2017 guidelines, infliximab and adalimumab were recommended both in monotherapy and in combination with thiopurines. However, from 2022 onwards, these anti-TNFα antagonists were recommended only in monotherapy mode [14,15].

The situation is slightly different for Crohn’s disease. ECCO guidelines from 2020 and 2024 were consulted. For patients with mild-to-moderate CD, budesonide is recommended as the first-line treatment for the induction of remission. In contrast, a broader range of pharmacological options is available for moderate-to-severe CD. Systemic corticosteroids are employed for induction of remission, whereas thiopurines are primarily used for maintenance. Additional agents with efficacy in both induction and maintenance include methotrexate, infliximab (as monotherapy or in combination with thiopurines), adalimumab (as monotherapy or in combination with thiopurines), certolizumab, ustekinumab, risankizumab, vedolizumab, and upadacitinib [16,17].

Table 4 provides a comprehensive overview of all the pharmaceutical agents discussed above. It is important to recognize the adverse effects of the pharmaceutical products used on IBD patients. Many of these drugs can cause serious complications, some even increasing the risk of cancer, so the choice of treatment must be weighed carefully. However, not all the drugs listed in this table are fully understood: in some cases, both their mechanisms of action and side effects remain unclear. For instance, 5-ASA, a first line drug used in many IBD patients, works in a way that is still not completely clear. Moreover, most of the biologics are relatively new, and, for this reason, some of their adverse effects may not yet be fully identified. Nonetheless, even in cases where an elevated risk of developing cancer is associated with a drug, the actual incidence is considered rare or very rare.

Although not included in the ECCO guidelines for therapeutic strategies for IBD, an additional class of drugs used in the treatment of ulcerative colitis and Crohn’s disease is represented by sphingosine-1-phosphate (S1P) receptor modulators. S1P is a lipid chemoattractant involved in the egress of lymphocytes from lymph nodes, and its receptor is expressed on lymphocytes. Thus, the modulation of S1PR prevents lymphocyte trafficking to sites of inflammation. There are currently two drugs of this class used in inflammatory bowel disease: ozanimod and etrasimod [20].

## 2. IBD-Related Cancer: Discovery of Biomarkers and Technological Approaches

### 2.1. The Medical Problem

#### 2.1.1. IBD and Cancer: A Long-Known Correlation

The correlation between cancer and inflammatory bowel disease has been known for almost a century. The first article on this topic dates back to the 1920s: J. Arnold Bargen published a paper titled “Chronic ulcerative colitis associated with malignant disease” [21] in 1928. Of course, this correlation was seen other times during the 20th century, but the studies investigating the connection between IBD and cancer incidence sometimes lacked statistical credibility. This correlation is, in fact, very hard to study, or, at least, to study without any bias: CD and UC patients had much more frequent contact with the healthcare system, so there was a higher probability of being diagnosed with cancer, or of being diagnosed earlier in life (this is known as surveillance bias). In addition, due to the complexity of cancer and to the differences between various types of cancer, forming correct comparison groups was difficult. Nonetheless, the correlation between IBD and malignancies was observed many times in the last century: patients with IBD were considered at increased risk for colorectal cancer in addition to breast, lung, small intestine, bile duct, and hematopoietic malignancies (such as leukemia and lymphoma) [22].

In the last few decades, a better understanding of cancer and IBD-related cancer has allowed researchers to carry out more reliable observational studies. IBD-related malignancies can be inflammatory-related or immunosuppression-related. The former refers to malignancies that arise due to damage caused by chronic inflammation: this kind of IBD-related malignancy consists mainly of gastrointestinal (GI) tract cancers. The latter case includes all cancers that develop due to treatment with immunomodulators, particularly immunosuppressive pharmaceuticals. For instance, a suppression of the immune system could prevent T cells from recognizing tumor cells, promoting carcinogenesis. Inflammation-related cancers can be overcome (by controlling inflammation itself), but this is not the case for treatment-related cancers, since other drugs should be used to protect IBD patients from risk, which is not always possible. However, not all immunosuppressive agents are linked to carcinogenesis: 5-ASA, for instance, has not been considered as a cancer risk increasing drug [23].

#### 2.1.2. Recent Observational Studies

Many observational studies have been performed to better understand not only the correlation (in terms of incidence and mortality in the IBD population compared to the general population), but also to understand risk factors.

An observational study published in 2019, the SIBDCS (Swiss IBD Cohort Study), analyzed 3119 IBD patients from Switzerland to assess whether they developed cancer and understand the possible risk factors. Malignancies were found in 3.9% (122) of patients. The detected malignancies were: 25.4% GI carcinoma, 22.1% GI dysplasia, 9.0% skin, 7,4% breast, 6.6% intestinal lymphoma, 3.3% extraintestinal lymphoma, 3% biliary cancer (the remaining were other cancers like leukemia, lung cancer, urogenital tract cancer, etc., …). Of the 122 patients with malignancy, cancer was observed in 81 patients over 5 years of follow-up (2.6% of the total cohort) [23].

The largest identified risk factors were age and recent use of immunomodulators (azathioprine). Moreover, malignancies were more frequently observed in males, in individuals diagnosed at an older age, and in patients with a longer duration of IBD. Treatment with antibiotics or steroids was also considered a risk factor, as were intestinal surgery and the presence of fistulas or abscesses, regarding intestinal malignancies. Along with risk factors, some protective elements were also found in 5-ASA use, biologics and stenosis.

The SIBDCS did not demonstrate an increased risk for overall cancer, but it reported a higher risk for lymphoma and biliary cancer (SIRs: 2.79 for Non-Hodgkin lymphoma, 2.98 for lymphoma overall, and 6.3 for biliary tract cancer), while no increased risk for colorectal cancer (CRC) or small bowel cancer (SBC) was observed. This absence of association with CRC may reflect the declining incidence attributed to improved screening guidelines and novel diagnostic tools. Specifically, Standardized Incidence Ratios (SIRs), a statistical and epidemiological measure comparing the number of cases of a disease in a study population with that expected in the general population (or another population of reference), were obtained [23]:

(1) $$SIR=\frac{Observed\;cases}{Expected\;cases}$$

In 2020, another study was conducted to analyze possible risk factors in IBD patients. In a cohort of 1209 IBD patients, 403 individuals were diagnosed with cancer in a 6-year follow up. As in the former study, the most frequent cancers were the ones concerning the digestive tract (32%). Other malignancies involved skin (14.9%), urinary tract (9.7%), lung (6.9%), genital tract (6.5%), breast (5.5%), thyroid (1.9%), lymphoma (2.7%), small bowel adenocarcinoma (SBA) (3.9%).

Overall, compared with the SIBDCS, this study also identified digestive tract malignancies as the most frequent but confirmed that CRC risk was not increased, while reporting SBA and lymphoma cases among the most relevant associations.

The study highlighted the following risk factors: localization of IBD (since UC frequently led to CRC and CD led to SBA), phenotype of IBD (extensive UC and perforating CD are more associated with malignancies), abdominal surgery, and disease duration. Interestingly, thiopurines and TNFα antagonists were not considered risk factors in this study [24].

Regarding these two pharmaceutical products, thiopurines were first used in the treatment of acute lymphocytic leukemia (ALL), but they are currently used for autoimmune diseases as well. They are immunosuppressors that are converted in the organism in active metabolites, particularly 6-thioguanine nucleotides (6-TGN), which are incorporated in DNA and RNA, causing the death of B and T cells (which explains why they are also effective in ALL treatment). Carcinogenesis is one of the known adverse effects of thiopurines: they are correlated with lymphoma and skin cancer. In fact, 6-TGN in the DNA leads to the activation of cellular mismatch repair (MMR) mechanisms that can cause mutations in the genome. In addition, 6-TGN is highly reactive to UV-A light, and this can cause production of reactive oxygen species leading to oxidative stress: this is specifically why skin cancer is more common in people treated with thiopurines [25].

Anti-TNFα agents are biologic pharmaceutical products (often monoclonal antibodies) that bind with elevated specificity to the proinflammatory cytokine Tumor Necrosis Factor α. These drugs are used in many autoimmune diseases such as psoriasis, rheumatoid arthritis, ankylosing spondylitis, and inflammatory bowel diseases. TNFα antagonists are widely used because of their high efficacy in decreasing inflammation. Nevertheless, they could also be correlated to carcinogenesis, although data are still inconsistent. In fact, blocked TNFα (and therefore the impairment of its biological action) could prevent the cells of the immune system from detecting cancer cells. However, the scientific community has not yet reached consensus on this matter, and further studies are certainly necessary [26].

Another research group used data from the UK Biobank to investigate the association between inflammatory bowel diseases (IBD) and cancer. This prospective cohort study demonstrated that individuals with IBD have an increased overall cancer risk, with a higher incidence observed particularly for specific malignancies such as gastrointestinal cancers, non-melanoma skin cancers, and cancers of the genital tract [27].

#### 2.1.3. Statistical Correlation Between IBD and Cancer Development

Although many cancer types have been diagnosed in patients with IBD, not all of them are considered related to the disease. To determine whether a cancer type is associated with IBD, it is important to examine the Standardized Incidence Ratios (SIRs), or fold change in cancer development risk, which indicate the incidence of a specific cancer in IBD patients compared to the general population. A high number of IBD patients diagnosed with a common cancer does not necessarily imply an increased risk, while it may simply reflect the cancer’s high prevalence in the general population. This explains why, the number of breast and lung cancer cases were higher than other tumors in some studies, but no significant correlation with IBD was found. Information on the incidence of different cancer types can be found in Table 5.

### 2.2. Proteomics Relevance in Personalized Medicine

Recently, the omics disciplines have gained significant relevance in the scientific context. Among these, proteomic aims to study proteomes, made up of all the proteins of a cell, a tissue, or an organism. Analyzing the proteome not only entails understanding the composition in terms of proteins, but also different protein isoforms, post-translational modifications, structures, multimeric complexes, protein–protein interaction dynamics [28].

Furthermore, proteomics provides information on the expression levels and alterations of individual proteins, precisely defining their roles within a biological matrix in both spatial and temporal dimensions. Nowadays, precision medicine (or personalized medicine) is becoming the new goal in therapy and proteomics is a key tool for this process. It is generally agreed that one-size-fits-all science will not to solve unmet medical needs completely. The aim of precision medicine is in fact to provide patients with customized treatments by taking into consideration genetics and epigenetics, physiology, environmental factors, disease progression, disease subtypes (more precise diagnosis), and medical history.

Genomic analysis has already started to be implemented in clinics (diagnosis, prognosis, pharmacogenomics), and many other fields (such as agriculture, industry, ecology, etc.). Personalized proteomics could exert a key role in precision diagnostic, but also in determining patient prognosis.

Currently, the greatest focus concerning clinical proteomics is on protein biomarkers discovery, i.e., biomolecules with an altered expression or presence that correlate their state with the presence of a disease and can be used to diagnose a pathology in patients. They can be either diagnostic or prognostic biomarkers. These have attracted significant attention especially in oncology, where reaching precise and early diagnosis is of the utmost importance. In fact, protein biomarkers can not only correctly identify a tumor but they can also be used to screen for the presence of a tumor at its initial stage, thereby enhancing the efficacy of therapy. Moreover, proteomics prognostic biomarkers are useful to assess disease severity and progression [29].

Biomarkers can be detected through different proteomic methods. The gold standard was initially 2D gel electrophoresis, but nowadays the most explored approaches are ELISA [30] and mass spectrometry (MS) [31], which are much more sensitive than the previous techniques and allow more precise quantitative studies of biomarker screening [32]. ELISA assays, while sensitive, are limited by antibody availability, high costs, and potential cross-reactivity, which can compromise specificity and simultaneous detection. These drawbacks can be overcome by mass spectrometry, which offers higher throughput, broader analyte coverage, and improved specificity without reliance on antibodies. MS-based proteomics use mass spectrometers to measure mass-to-charge values of peptides to analyze multiple proteins.

For instance, mass spectrometry-based proteomics has emerged as a powerful tool for developing diagnostic peptide panels by enabling high-resolution identification of disease-associated molecular signatures. In an initial study by Frantzi et al., capillary electrophoresis coupled to mass spectrometry (ESI-TOF MS) was applied to urinary samples, resulting in an 86-peptide classifier trained on 108 subjects (40 renal cell carcinoma patients and 68 controls) and validated on an independent cohort of 76 samples; robust specificity was further demonstrated through external cross-validation on over 1000 samples from patients with various renal and urological conditions (80% sensitivity, 87% specificity in validation) [33]. Subsequently, the same group conducted a large, multicenter validation study involving a total of 1357 participants: 721 with primary urothelial bladder cancer and 636 with recurrent disease. Two peptide panels (116 and 106 peptides) were developed using nested cross-sectional designs (n = 451 primary; n = 425 recurrence), and then tested in independent validation sets (270 primary, 211 recurrent), achieving AUCs of 0.87 and 0.75, respectively. At optimal thresholds, the primary cancer panel reached 91% sensitivity and 68% specificity, whereas the recurrence panel achieved 87% sensitivity and 51% specificity [34].

Another example of mass spectrometry-based proteomics was provided by Njoku et al. in 2023 [35]. This group used SWATH-MS to develop multi-marker diagnostic panels in cancer detection. In a prospective case–control study, urine samples were collected from 104 symptomatic post-menopausal women (50 with histologically confirmed endometrial cancer and 54 control cases) and analyzed using SWATH-MS coupled with machine learning to identify discriminatory protein signatures. Features with individual predictive value (AUC > 0.70) were combined via logistic regression into a 10 markers panel which achieved an impressive AUC of 0.92 (95% CI: 0.86–0.97), with sensitivity of 83.7% and specificity of 83.9%. Notably, this signature also demonstrated excellent discrimination for early-stage disease (AUC 0.92 [0.86–0.90]).

In an effort to investigate the molecular alterations associated with cervical cancer, Xu et al. conducted a proteomic study on exfoliated cervical cells from cancer patients and healthy controls, so as to define disease-specific protein signatures with both mechanistic and diagnostic relevance. Distinct from previous examples based on label-free quantification, this work employed a tandem mass tag (TMT)-based labeling strategy, which allows multiplexing of samples through isobaric tagging and simultaneous analysis by LC-MS/MS. Through this approach, the authors identified 351 differentially expressed proteins, including 247 upregulated and 104 downregulated in cervical cancer samples, which were functionally enriched in pathways such as PI3K–Akt signaling, extracellular matrix–receptor interactions, and complement and coagulation cascades. Functional annotation indicated a strong association with exosome-derived proteins and regulators of apoptosis, highlighting peroxiredoxin-2 as a candidate with potential carcinogenic activity. This study exemplifies how label-based mass spectrometry provides the sensitivity, reproducibility, and breadth required to capture cancer-associated proteomic alterations [36].

A further interesting approach was that of Whiteaker et al., which introduced a comprehensive pipeline for the verification of plasma biomarkers using targeted proteomics. Specifically, they employed Multiple Reaction Monitoring (MRM) mass spectrometry. This approach addresses the challenge of validating numerous candidate biomarkers identified through high-throughput technologies. In their study, the researchers began with more than 1000 candidate biomarkers identified from a mouse model of breast cancer. Through a data-dependent triage process, they prioritized a subset of these candidates for further validation. Subsequently, they developed 88 novel quantitative assays based on MRM MS. These assays were performed simultaneously and evaluated in plasma samples from 80 animals. The results confirmed that 36 proteins were elevated in the plasma of tumor-bearing animals, validating their potential as biomarkers for breast cancer. This study demonstrates the efficacy of MRM-based targeted proteomics in the verification of plasma biomarkers, providing a scalable and efficient method for biomarker validation in clinical research, especially since this technique allows multiple samples to be analyzed at once [37].

Although the four cases discussed do not concern IBD-related cancer, they provide important information regarding the potential role of MS in future personalized medicine in all cancers.

### 2.3. Most-Used Proteomics Techniques in Clinical Studies

**Figure 2:**
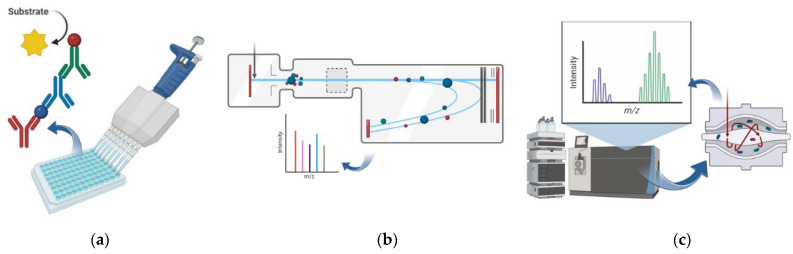
Overview of multi-protein analysis techniques: (**a**) ELISA; (**b**) TOF-MS; (**c**) Orbitrap-MS.

Mass spectrometry is not the only proteomic technique. An essential tool in the analysis of proteins is represented by immunoassays, which are assays based on the recognition of target proteins by antibodies. One of the most used immunoassays is ELISA (Enzyme-Linked ImmunoSorbent Assay, Figure 2a). Target proteins can be detected by a primary antibody, which is recognized by a secondary antibody, engineered with an enzymatic function that allows protein detection (such as horse-radish peroxidase domain that generates fluorescence) [30]. Novel immunoassay techniques have also been developed for the identification of cancer biomarkers: this is the case of the Leucine-rich α2 glycoprotein (LRG) detection kit based on a latex turbidimetric immunoassay, in which specific antibodies attached to a latex bead can detect a serum cancer biomarker [38].

Antibody-based proteomics is still relevant nowadays particularly in hospital diagnostic routines, but its greatest limitation is the need for specific antibodies against selected biomarkers. These techniques cannot be used to discover novel biomarkers.

By contrast, mass spectrometry offers higher sensitivity and specificity and is therefore the most convenient proteomic approach. The most common analyzers include time-of-flight (TOF, Figure 2b) and Orbitrap instruments (Figure 2c). TOF determines peptide mass based on ion travel time, while the orbitrap detects frequency of ion motion, both generating high-resolution mass spectra.

Ionization methods typically include electrospray ionization (ESI), matrix-assisted laser desorption/ionization (MALDI), and surface-enhanced laser desorption/ionization (SELDI). While MALDI and SELDI both rely on laser-induced ionization, SELDI adds molecular selectivity through functionalized surfaces, which is useful for comparative studies [31,39].

MS-based proteomic analysis of a biological sample can follow two approaches: targeted MS and untargeted MS. Targeted MS is used to recognize specific proteins, whereas the untargeted approach is used to identify all the proteins inside a specimen, potentially allowing novel biomarkers identification [31].

A common workflow for MS-based proteomics is the bottom-up approach, in which proteins are extracted, digested (e.g., with trypsin), and analyzed by LC-MS/MS (Liquid Chromatography-Tandem Mass Spectrometry). Peptides are separated by liquid chromatography, ionized, and introduced into the spectrometer to obtain mass-to-charge (*m*/*z*) values (MS1). Fragmentation followed by a second stage of MS (MS2) then allows peptide sequencing and protein identification [31].

Furthermore, MS analysis can exploit a data-dependent (DDA) or data-independent (DIA) acquisition. On the one hand, in DDA MS, precursor ions that likely correspond to peptides are selected based on MS1 data and subjected to MS2 fragmentation. This means that MS2 analysis depends on the information collected in MS1. In contrast, in DIA all ions within a predefined *m*/*z* range are systematically selected for fragmentation, regardless of their intensity and identity. An example of DIA approach is the SWATH-MS analysis, where the entire ion population is fragmented in sequential windows to ensure comprehensive coverage [31].

### 2.4. Proteomic Approaches in IBD

Proteomic application in the field of inflammatory bowel disease is clinically relevant from different points of view. Firstly, proteomics has been shown to be effective in diagnosing different IBD subtypes. Secondly, proteomics can be used to find predictive hallmarks of IBD-related cancer.

In other words, proteomic techniques are currently being used to better understand all aspects of IBD. For instance, in a Swedish study by Salomon and collaborators, proteomics was used to characterize protein profiles of different IBD subtypes: they were able to distinguish UC and CD protein expression profiles from healthy controls. Immunochemistry assays were performed to discriminate between CD and UC inflammation-related protein expression patterns. However, there were many proteins altered in both UC and CD, creating a sort of spectrum of IBD. This changes the way IBD was seen up until the last few years [40].

Another study published in 2020 used proteomics techniques to compare IBD protein content in the gut with control groups (exploratory cohort: 34 controls, 72 CD, 56 UC, 5 unclassified; validation cohort: 28 controls, 27 CD, 15 UC). The authors used MALDI-TOF/MS to analyze peptides and Orbitrap MS to analyze proteins, analysis was conducted in blind mode. Spectra obtained were used to distinguish an exploratory control group and the IBD cohort (through an algorithm), and the two groups were discriminated with 81% sensitivity and 97% specificity. The findings were later confirmed in a validation cohort (even though in this case sensitivity was lower, 55%). This research group not only found that MS analysis outperformed fecal calprotectin in diagnosing IBD, they also found which proteins were overexpressed in the pathology: immunoglobulins, neutrophil proteins. Furthermore, downregulated proteins were found to be linked to nucleic acid assembly and cancer risk. Thus, this study highlights the potential of stool peptidomics and proteomics in improving IBD diagnosis and understanding disease mechanisms. What is more, downregulated proteins correlated to cancer could also be used for cancer risk evaluation in IBD patients [41].

Apart from learning more about the disease, proteomics can also facilitate preventing carcinogenesis, one of the gravest IBD complications. Mass spectrometry has been used to identify novel cancer biomarkers which, when detected, enable patients to start early oncologic treatment. As a result, they have a greater chance of overcoming cancer. Therefore, proteomics has already started to gain a significant role in precision medicine for IBD, especially MS-based proteomics like MALDI-TOF and SELDI-TOF. These methods are useful to quantify proteins from biological specimens, and to compare expression patterns between ill patients and healthy controls as well [42]. Thus, this study highlights the potential of stool peptidome and proteome analysis to improve IBD diagnosis and clarify disease mechanisms.

Since IBD manifests in the digestive tract, the search for biomarkers is often directed to stool specimens. The prevalent fecal biomarkers that indicate intestinal inflammation are lactoferrin and calprotectin [43]. However, since severe diarrhea can sometimes prevent patients from obtaining representative stool samples, biomarkers from biological fluids started being investigated, in order to enhance compliance. These can derive from fluids such as serum, plasma, urine, cerebrospinal fluid, and they allow easy and noninvasive analysis. The best example of a serum biomarker in IBD is the C-reactive protein, but novel and more specific biomarkers are currently being researched [44].

As already affirmed, prior to the widespread implementation of mass spectrometry and the advent of next-generation proteomics, a substantial proportion of proteomics relied on the ELISA approach. Nevertheless, these methods presented intrinsic limitations, particularly due to their dependence on prior knowledge of the target protein and the availability of specific antibodies, thereby restricting their applicability in large-scale or unbiased biomarker discovery. To emphasize these aspects, the following two examples will be mentioned.

In 2006, Tatsumi et al. [45] investigated protein expression in UC-associated colorectal neoplasms, focusing on two cytoskeletal proteins, cytokeratin 7 and 20 (CK7 and CK20). To assess their expression, the authors employed monoclonal antibodies specific for these proteins. This approach was feasible since previous studies had already established that CK7 is expressed in the colonic epithelium under neoplastic conditions, but not in physiological states. The reliance on monoclonal antibodies, in fact, represents a limitation in biomarker discovery: as in proteomics techniques such as ELISA, their use is restricted to known targets.

A similar study was conducted by Hashimoto et al. in 2013 [46], who quantitatively evaluated the expression levels of the chemokine CCL20 in the rectal mucosa of ulcerative colitis patients compared with healthy controls. Using immunohistochemical assays with monoclonal antibodies, they demonstrated significantly higher levels of CCL20 in UC patients. Again, this approach was possible because altered expression of CCL20 in UC mucosa had already been described in prior studies.

While these examples cannot be classified as true proteomics studies, since they only targeted a limited number of proteins, they illustrate a key limitation shared by immunohistochemistry and ELISA-based proteomics: both require prior knowledge of the target and the availability of specific antibodies. This restriction poses a significant challenge in comprehensive proteomics analyses, where hundreds or thousands of proteins are examined simultaneously. In such contexts, the high cost and impracticality of generating monoclonal antibodies for each protein severely constrain large-scale biomarker discovery.

In contrast, proteomics approaches based on mass spectrometry have revolutionized the field by allowing high-throughput analysis of complex proteomes without the prerequisite of specific antibodies. For instance, Yeo and collaborators in 2006 compared protein expression profiles in the colonic mucosa of two groups: mice with dextran sodium sulfate (DSS) induced colitis, which had developed colorectal cancer, and healthy controls. With a 2D electrophoresis they discovered 38 differentially expressed proteins, 27 of these were then identified with MALDI-TOF mass spectrometry. The results demonstrated that one protein, transgelin, was down-regulated in mice with induced colorectal cancer. Moreover, the MALDI-TOF analysis showed that two different isoforms of transgelin were present in the colorectal cancer group. In fact, MS facilitates the characterization of post-translational modifications and protein isoforms: this would most certainly be more challenging to assess with antibody-based methods [47].

Another group of research in 2011 used an MS-based proteomic approach in order to better understand the progression from ulcerative colitis to colorectal cancer. In this case, a group of UC progressors (who tend to develop cancer) was compared to a group of UC non-progressors. Colonic biopsies were gathered to carry out analysis of proteomes with Orbitrap MS, enabling quantitative profiling of more than 1700 protein groups. The analysis revealed distinct proteomic signatures in both dysplastic and non-dysplastic tissues of UC progressors, with major alterations in mitochondrial proteins, cytoskeletal elements, RAS signaling, apoptosis-related proteins, and metabolic pathways [48].

In order to better outline features of different proteomic approaches, we propose Table 6, which contains advantages and disadvantages concerning immunoassays and mass spectrometry methods.

## 3. Proteomics Application in IBD-Related Cancer Studies

### 3.1. Inflammation-Related Cancer

Inflammation-related malignancies arise from inflammatory processes and metabolites produced as a result of inflammation. Many of these metabolites, such as reactive oxygen species (ROS), are highly reactive and can damage various cellular components, including DNA. These types of cancers are considered direct consequences of inflammation and therefore tend to develop in chronically inflamed tissues. In the context of inflammatory bowel diseases (IBD), this means that inflammation-related cancers predominantly affect the gastrointestinal tract [49].

#### 3.1.1. Colorectal Cancer

IBD-associated colorectal cancer is characterized by molecular, histologic and endoscopic features that differentiate it from sporadic CRC [50].

Among all cancer types, the correlation between colorectal cancer (CRC) and IBD is one of the most extensively and longest-studied associations. In 2001, a meta-analysis described the incidence of CRC in IBD patients, highlighting how the risk of CRC increased in UC patients in connection to disease duration. This meant that the risk of developing CRC was cumulative: in particular, the increment of the risk was 2% at 10 years of disease duration, 8% at 20 years, 18% at 30 years [51].

However, a population-based study (1976–2008) observed CRC incidence in the general population comparing it to that in the IBD population. Out of 19,413 CRC cases, 38 (0.2%) patients had a prior IBD diagnosis, with a mean age of diagnosis inferior in IBD than in the general population (56.9 vs. 70.9 years; *p* < 0.001) [52]. Additionally, a following population-based study (1996–2011) from the Utah Cancer Registry found that among 12,578 cases of CRC, 101 (0.8%) had a prior IBD diagnosis. The mean age of non-IBD patients was greater at time of CRC diagnosis than in those with IBD (67.1 vs. 52.8 years; *p* < 0.001) [50]. In both population-based studies UC patients were more at risk for CRC than CD patients.

Another group of researchers in Australia took data from the cohort of an IBD study (1977–1992) and collected new follow-up data for 14 years. This confirmed that the actual incidence of CRC is lower than that reported in 2001. The cumulative incidence reported for UC patients was 1% at 10 years of disease duration, 3% at 20 years, 7% at 30 years and for CD it was even lower (1% at 10 years, 1% at 20 years, 2% at 30 years) [53]. This confirms what has already been said about the decreasing risk for CRC in IBD patients. In fact, for patients with UC there is an actual 6.3-fold increased risk after 30 years of disease duration, and for CD patients it is lower (1.8-fold increased risk).

Even though colorectal cancer is the third most common cancer worldwide, according to the National Cancer Institute, there are still no protein biomarkers available to screen patients in clinics. At present, screenings and check-ups consist of endoscopies, fecal blood tests, and DNA stool tests. While the last two options might not be invasive, collecting stool samples can be challenging for IBD patients, as already outlined.

Furthermore, patients diagnosed through fecal hemoglobin testing and colonoscopy are often diagnosed too late, precluding the possibility of early surgical colon resection, despite its proven curative potential. The search for serum biomarkers started for this reason. A study by Ahn et al., in 2019 [54], used plasma samples to characterize the protein expression pattern in different stages of CRC. They used SWATH-MS and detected 37 differentially expressed proteins. Out of these, 7 candidates were corroborated using Western blot and ELISA (CST3, GPX3, CFD, MRC1, COMP, PON1 and ADAMDEC1).

Within these, the protein Paraoxonase 1 (PON1), an extracellular enzyme for detoxification, immunomodulation and antioxidation in the intestine, had already been found to be downregulated in IBD patients (both CD and UC) in 2009. Consequently, its downregulation was already known to be linked with intestinal inflammatory activity [55]. Nevertheless, it should be noted that this correlation has been tested only in a limited number of studies, and further validation is required before PON1 may be considered a reliable clinical biomarker. As previously mentioned, although numerous biomarker studies exist for colorectal cancer, no protein biomarkers are currently used in routine clinical practice.

PON1 is a member of the paraoxonase family (PON1-3), a group of proteins known for their anti-oxidant properties. PON1 is secreted by cells and can interact with high-density lipoproteins giving them anti-oxidant and anti-inflammatory properties. However, PON gene loci have been associated both with inflammatory diseases (e.g., atherosclerosis, Alzheimer’s) and cancer. Lower expression of PON1 could in fact determine a less effective response to inflammation [56].

At any rate, the correlation between PON1 and IBD inflammatory activity was later confirmed by a meta-analysis in 2024. Its aim was to analyze oxidative stress-related markers: plasma concentration of PON1 was lower in IBD patients [57].

Therefore, PON1 could be used as a marker for disease activity and, consequently, a marker for colorectal cancer risk as well, provided that subsequent studies demonstrate its ability to detect CRC with high sensitivity and specificity.

Another study used MALDI-TOF MS and Orbitrap MS to analyze salivary samples of peptides and proteins, respectively, in CD, UC, CRC and controls. In IBD and CRC groups, 73 proteins were found that were absent in controls. These proteins were mainly involved in adaptive and innate immunity. This group also analyzed stool samples with the same proteomics approaches and found 30 human proteins, 4 present only in CD. Out of these 4, 2 proteins, Basic Salivary Proline-Rich Protein 1 (PRB1) and Salivary Acidic Proline-rich Phosphoprotein, are of salivary origin.

Peptide fragments of PRB1 (GQ-17 and GG-17) were also tested in vitro on CRC HT-29 cell line and it was observed that GG-17 had a role in cellular proliferation, suggesting its potential role as a CRC biomarker [58].

In physiological conditions, basic salivary proline-rich proteins need to be cleaved in order to go from the precursor to the activated state. In fact, they usually play a role in the precipitation of dietary tannins (polyphenolic compounds deriving from plants). Yet, some fragments of PRB1 could be involved in carcinogenesis (e.g., the GG-17 fragment may act as an inducer of cellular proliferation) [59].

#### 3.1.2. Small Bowel Cancer

Small bowel cancer (SBC) is a rare condition that often affects the ileum, but it can also develop in the duodenum and the jejunum. Since CD is often located in the small intestine, the correlation between chronic inflammation and malignancy in that area was assessed.

A population-based study published in 2017 analyzed the entire population of Denmark (with an age ≥ 16 years) from 1978 to 2010: during this study 20,917 patients with CD and 42,872 patients with UC were followed, and 40 patients with IBD-SBC were identified. Out of 40, 23 cases were CD patients with Small Bowel Adenocarcinoma (SBA). The other cases concerned other small bowel cancer types. With this analysis, a 14-fold increased risk of developing SBA (the most common type of SBC) was demonstrated in CD patients, whereas UC patients did not seem to be at higher risk [60].

Early detection of small bowel cancer is challenging, and there is no standardized procedure to screen small bowel lesions (predictive of cancer). That is also because they cannot always be detected through ileocolonoscopy. In this context, a biomarker would be of crucial importance, since it would allow a less invasive and more efficient screening.

The gold standards in recent decades were C-reactive protein (CRP) and faecal calprotectin (FC), but their specificity for small bowel cancer was low. Consequently, the need for a novel, more specific biomarker led to the discovery of Leucine-rich α2 glycoprotein (LRG) profile expression, which assesses disease activity of IBD. A study conducted by Asonuma et al. compared the usefulness of LRG, CRP and FC as serum (in the first two cases) and stool (in the last case) biomarkers. LRG levels of expression were quantified through latex turbidimetric immunoassay. Their conclusion was that LRG was the best out of the three proteins in determining small bowel inflammation [38]. This protein is involved in many processes, such as angiogenesis, apoptosis inhibition, and the cellular epithelial–mesenchymal transition (a common biological step that can lead to metastasis) [61].

The utility of LRG as a biomarker was recognized in 2020, since this protein was approved in Japan as a biomarker for assessing activity in UC and CD. Its presence, even though it is not strictly related to the small bowel, can show whether the mucosa of the intestine is healing or not: if the damage to the bowel wall persists, the risk of cancer is increased. Thus, LRG could be investigated as an indirect predictive small bowel cancer biomarker, pending validation in future studies to confirm its sensitivity and specificity [62].

#### 3.1.3. Cholangiocarcinoma

Although cholangiocarcinoma (CCA) is a rare cancer, its mortality is one of the highest: only 22% of patients diagnosed with CCA in the US survive after 5 years. It does not affect the digestive tract directly since it develops in the bile ducts. The problem with this cancer is that it does not present very intense or specific symptoms, so when it is diagnosed it is often too late for the patient. In this case knowing risk factors would be helpful, especially in the field of prevention.

One risk factor for CCA is IBD itself: compared to the general population, IBD-patients are more at risk of developing cholangiocarcinoma. A meta-analysis of multiple population-based studies found that patients affected by IBD (both UC and CD) have a 2.61-fold increased risk in intrahepatic cholangiocarcinoma, and a 1.47-fold increased risk of extrahepatic cholangiocarcinoma [63].

Another factor influencing the development of this rare cancer is the presence of concomitant Primary Sclerosing Cholangitis (PSC). This is a condition with an unclear etiology that leads to the formation of fibrotic biliary strictures and can also progress to liver cirrhosis. PSC is strongly related to IBD: about 60–80% of individuals diagnosed with PSC have a prior diagnosis of IBD (and 2–5% of IBD patients develop PSC). In a population-based cohort study conducted on hospital databases in Norway and the National Patient Register in Sweden, 141,960 IBD patients were observed (3.2% of them had concomitant PSC). In a median follow-up of 10 years, relative risk for CCA was assessed, and it was found that PSC-IBD patients have a 140-fold increased risk of developing biliary tract cancers, with the highest risk for intrahepatic cholangiocarcinoma (220-fold risk) [64].

As previously stated, cholangiocarcinoma (CCA) is a very aggressive cancer, but as often happens with liver-related pathologies, symptoms are not present until late disease state. Specific and non-invasive tests are therefore necessary.

There are two biomarkers used for CCA diagnosis: Carcinoembryonic Antigen (CEA) and Carbohydrate Antigen 19-9 (CA19-9). CEA is a protein normally found in embryonic tissues; low levels persist in adults, but elevated concentrations may indicate carcinogenesis. However, it is not a specific biomarker. The second marker is CA19-9: this is not a protein, but an increase in its levels indicated a correlation to primary sclerosing cholangitis and CCA. Specifically, CA19-9 level of >75 U/mL had 99% specificity and 69.5% sensitivity (serum CA19-9 levels were measured in 192 UC patients) in the diagnosis of PSC (PSC diagnosis was validated by magnetic resonance cholangiopancreatography) in IBD patients without CCA, so it is currently used as an early biomarker [65,66].

CA19-9 is a glycosylating agent with many substrates. Therefore, its activity can lead to hyperactivation of some pathways (e.g., the EGFR intracellular pathway), thus leading to carcinogenesis. Moreover, it can promote metastasis (since tumor cells that express CA19-9 on the plasmatic membrane can interact with E-selectins of blood vessels) [67].

Nevertheless, biomarkers of protein nature have also been explored: this could be used in combination with CA19-9 to obtain a more specific action. In 2013, a study aimed to identify novel biomarkers by using proteomic techniques such as 2-D gel electrophoresis and MALDI-TOF MS, by comparing protein expression patterns in CCA tissues and healthy tissues. Particularly, chaperonin-containing TCP1 subunit γ (CCTγ) and S100 calcium-binding protein A9 (S100A9) were overexpressed in tumors compared to controls in MS analysis. Moreover, these results were validated with immunochemistry methods [68]. This shows that these two proteins could be used as biomarkers for CCA, along with CA19-9. On the one hand, CCTγ is a component of a 16-subunit chaperonin complex that plays a crucial role in the proper synthesis and folding of actin and tubulin. Similarly to heat shock proteins, overexpression of this chaperonin may indicate that the cell is under stress or in an unhealthy state [69]. On the other hand, S100A9 is a calcium-binding protein that, under inflammatory conditions, acts as a modulator of the inflammatory response by promoting white blood cell recruitment and inducing cytokine production and release. Therefore, in addition to being used as a biomarker, it also represents a potential therapeutic target, since inhibiting its activity leads to a reduction in the intensity of inflammation [70].

### 3.2. Immunosuppression-Related Cancer

The correlation between drugs used for chronic inflammation of the digestive tract and malignancy has been analyzed several times. The increased risk for cancer seems to relate to the assumption of thiopurines and tumor necrosis factor-α antagonists. Data about carcinogenesis caused by IBD therapies will be discussed in this section.

#### 3.2.1. Skin Cancer

Skin cancer can manifest itself in two main forms: melanoma and non-melanoma skin cancer (NMSC). The most common NMSC is basal cell carcinoma, while squamous cell carcinoma occurs with less frequency.

Skin cancer malignancy detection in IBD patients was investigated in a cohort of 108,597 patients with inflammatory bowel diseases, allowing a better understanding of risk factors. The results of this population-based study showed an incidence of melanoma of 57.1/100,000 in IBD, compared to 44.1/100,000 in the general population (60.8/100,000 in CD patients). With this data the standardized incidence ratio can be extracted: SIR found was 1.29 for IBD and 1.38 for CD. In the same study, the incidence of NMSC found was 912/100,000 in IBD, 623/100,000 in Non-IBD (SIR = 1.46). Therefore, the risk for NMSC is higher than that of melanoma. Moreover, the correlation between the incidence of skin cancer and the use of immunomodulators (thiopurines and anti-TNF biologics) was assessed. Since the 1990s, both the use of these medications and the number of melanoma and non-melanoma skin cancer diagnoses have increased. However, even though this temporal association suggests a possible link, it does not establish a causal relationship [71].

Another meta-analysis by Huang and colleagues investigated the incidence of skin cancers in IBD patients treated with thiopurines and without thiopurines. Although the efficacy of 6-mercaptopurine and azathioprine are good for IBD management (in particular when patients are resistant to corticosteroids), the results of this analysis showed an increased risk for skin cancer in thiopurine use, especially for NMSC. However, Huang and collaborators also found that there might be a correlation with geographic distribution as well, since they compared results obtained in North America and Africa. In fact, the most crucial risk factor for skin cancer is known to be sun exposure, so a synergistic effect between sunlight and thiopurines has therefore been inferred. In addition, no correlation between thiopurine use and melanoma skin cancer was found in this meta-analysis [72].

Treatment with biologics was also examined through a pooled analysis by Osterman et al. that investigated the correlation between the use of adalimumab (a TNF antagonist biological drug) and the increased risk of carcinogenesis in IBD patients. Moreover, they compared adalimumab monotherapy with adalimumab combination therapy with thiopurines. They gathered data from 1594 CD patients from the clinical trials of adalimumab. For these patients, malignancy was found in 34 people: 10 patients treated with adalimumab monotherapy, 24 patients treated with adalimumab combination therapy. The results led the authors to conclude that there is no increased risk of NMSC in patients receiving adalimumab monotherapy, whereas combination therapy is associated with a 5-fold increased risk [73].

According to the National Cancer Institute (NCI), visual exams conducted by doctors remain the standard procedure for skin cancer screening and diagnosis. If unusual moles or birthmarks or an abnormal pigmentation of the skin are present, further analysis consists in biopsy and histological analysis by a pathologist. There are no biomarkers available in clinic, but some studies have been carried out, especially regarding basal cell carcinoma (BCC), the most common type of skin cancer.

Berl and colleagues analyzed the protein expression pattern of BCC patients with mass spectrometry, finding which proteins were overexpressed. Results were later confirmed by mRNA quantitative analysis. This study found that protein profiles were symmetric to BCC subtypes division: each BCC subtype differentiated itself from other subtypes. This means that this kind of tumor is very complex and more studies will be needed to understand the heterogeneity of skin cancer subtypes and to discover novel diagnostic and prognostic biomarkers. Still, this study found two proteins that were overexpressed uniquely in nodular BCC subtype: cytochrome P450 (CYP2W1) and Neurotrophic Receptor Tyrosine Kinase 3 (NTRK3) [74].

Cytochrome CYP2W1 is normally expressed during fetal development, where it plays a role in intestinal maturation due to its tissue-specific expression. In cancer, however, it is also expressed in adults. It functions in metabolism, acting on both endogenous and exogenous substrates. Its oxidative activity can sometimes be harmful, as it may lead to the production of reactive oxygen species (ROS) [75].

Neurotrophic tyrosine receptor kinase 3 (NTRK3) belongs to the neurotrophic tyrosine receptor kinase (NTRK) family and recognizes neurotrophin-3 (NT-3) as its primary ligand. Oncogenic mutations in NTRK3 can result in constitutive activation of the receptor, leading to aberrant signal transduction pathways that drive carcinogenesis. Such alterations have been implicated in several malignancies, including pancreatic cancer [76].

Being able to associate a patient to a cancer subtype is essential for prognosis, but also to understand which therapy option will be most efficient (and, conversely, which will not work).

#### 3.2.2. Lymphoma

Treatment with thiopurines has also been investigated to understand its correlation with lymphoma, due to more-frequent-than-expected reports of lymphoproliferative disorders in IBD patients treated with 6-mercaptopurine and the correspondent prodrug.

In a prospective observational cohort study in France 19,486 IBD patients were observed from 2004 to 2007. 23 cases of lymphoproliferative disorders were detected: 1 Hodgkin’s lymphoma, 22 Non-Hodgkin’s lymphomas. The authors observed that the incidence of non-Hodgkin’s lymphoma was 0.90 per 1000 patient-years for those receiving thiopurines, 0.20 per 1000 for those who discontinued therapy, 0.26 per 1000 for those who never received thiopurines. Based on these results, the authors verified which of the patients used thiopurines, and determined that the risk for lymphoproliferative disorders (especially Non-Hodgkin’s lymphoma) was 5 times higher (5-fold increased risk) [77].

Some years later, a retrospective cohort study was published by Kahn and colleagues. They analyzed a cohort of 36,891 total patients with UC, 4734 of them (13%) were treated with thiopurines for a median time of 1 year. Out of the last-mentioned group the following incidences of lymphoma were found: 0.60 per 1000 patient-years for patients who never received thiopurines, 0.28 for patients who had discontinued treatment, 2.31 for patients who were receiving thiopurines. This data supported the statement that UC patients treated with thiopurines have a 4-fold increased risk of developing lymphoma, and that risk is decreased in patients who discontinued therapy [78]. These results are very similar to those in the previously mentioned study.

However, lymphoma might not solely be related to immunosuppression, it could also be induced by chronic inflammation itself. Many studies have investigated the role of an oncogenic virus, EBV (Epstein–Barr Virus), that can reside in B lymphocytes in physiological conditions. In cases of chronic inflammation, immunosuppression, and other impairments of the immune system, this virus can enter the lytic cycle, leading to B cell proliferation and potentially resulting in lymphoma, often diffuse large B-cell lymphoma (DLBL). For instance, a Dutch nationwide study investigated this correlation. In a cohort of 17,834 IBD patients, 44 had lymphoma. The expected number of lymphomas was 34.63, so the SIR was 1.27, which is not particularly elevated. More significant was the increased risk observed in age categories: 35–39 (SIR 9.32, 95% Confidence Interval: 3.89–17.07) and 45–49 (SIR 3.99, 95% Confidence Interval: 1.22–8.36). At any rate, of the 44 patients with lymphoma, 19 were exposed to thiopurines. Out of these 19, 15 were tested for EBV and 11 were EBV+. This suggested a correlation between EBV-related lymphoma and thiopurine therapy. Of 23 patients with lymphoma who were not exposed to thiopurines, 16 were tested for EBV and none was positive. For this reason, it is difficult to assess whether lymphoma is caused by chronic inflammation or immunosuppression and further investigation is necessary [79].

There is no standard procedure regarding lymphoma screening. For instance, the NCI states that for Non-Hodgkin’s Lymphoma the methods of cancer diagnosis are blood tests followed by CT (Computed Tomography) and PET (Positron Emission Tomography) scans, or potentially bone marrow aspiration and biopsies.

At any rate, studies to find protein biomarkers have also been conducted: in 2009, a study confirmed the relationship between diffuse large B cell lymphoma (the most common aggressive type) and the chemokine CXCL13 expression. The authors induced intestine inflammation in transgenic mice, finding that overexpressed CXCL13 induced an increase in B cell number and, additionally, promoted their mobilization in the intestinal lamina propria. Overexpression was validated with ELISA [80].

A longitudinal prospective study published in 2017 confirmed what had been previously discovered. Blood samples were taken 15 to 25 years before diagnosis of lymphoma, and concentration of proteins was measured. The authors found a strong association between the blood levels of CXCL13, sCD23, sCD27, sCD30 and the risk of developing lymphoma. The plasma concentration of CXCL13 and other lymphoma-related proteins was assessed with immunochemistry technologies. The only protein that was found to be associated with diffuse large B-cell lymphoma was CXCL13: its plasma levels increase over time, and, thus, it can be used as a biomarker [81].

CXCL13 is a chemokine primarily responsible for the recruitment of B lymphocytes. When CXCL13 interacts with its receptor CXCR5 (the CXCL13-CXCR5 axis), CXCR5 activates intracellular signaling by promoting the exchange of GDP for GTP on G proteins. This activation triggers downstream G protein-coupled receptor pathways, leading to aberrant signal transduction and the acquisition of hallmark tumor cell behaviors [82].

Concerning B-cells EBV-related lymphomas, a study was conducted on IBD patients exposed to anti-TNFα agents, assessing the presence of EBV by quantifying EBV genomes in peripheral blood. EBV genomes were higher in quantity in patients treated with TNFα antagonists compared with controls. This was in reality a genomics study, but it provided evidence for the necessity of further analysis of EBV proteome traces in the blood of at-risk patients. For instance, the concentration of two protein factors indicating transcription activation, ZEBRA and RTA, could be measured in future studies in order to assess their potential as lymphoma biomarkers [83].

#### 3.2.3. Glioblastoma

The gut–brain axis has become one of the most relevant topics of biomedical research in the last few decades. The brain and intestines are connected through hormones, the immune system and the nervous system, and this might be used to study possible correlations between gut and brain pathologies [84].

Since the connection between glioblastoma (GBM), the most aggressive and incurable malignancy of the brain, and IBD has not been investigated directly, the gut microbiome profiling may be used to make indirect assumptions.

In fact, glioblastoma has been shown to be characterized by microbiome dysbiosis. Ishaq and collaborators used fecal samples to profile the intestinal bacteria of GBM patients, comparing it to healthy controls. Results showed that GBM patients had higher microbial diversity. Specifically people with glioblastoma had an increase in Proteobacteria (Enterobacteriaceae, Bacteroidaceae, Lachnospiraceae) and a decrease in Firmicutes (Veillonellaceae, Rikenellaceae, Prevotellaceae). Moreover, opportunistic bacteria were more present in GBM, such as Escherichia coli, Bacteroides vulgates, Enterobacter aerogenes, Clostridium botulinum and Klebsiella oxytoca [85].

On the other hand, it is typical for IBD patients to showcase a less rich microbiome, with an increment of Actinobacteria, Fusobacteria and Proteobacteria (like adherent invasive *E. coli* strains) and a decrease in Bacteroidetes, Tenericutes and Verrucomicrobia [10].

This comparison suggested that there is no direct correlation between these two diseases. Nevertheless, the IBD-GBM correlation can assume significance when considering TNFα antagonists. In fact, a study in 2016 quantified the risk of glioblastoma in anti-TNF patients in terms of ROR (Reporting Odds Ratio), a statistical measure used in pharmacovigilance. This study used the FDA’s Adverse Event Reporting System (FAERS) and the World Health Organization (WHO) drug monitoring database. Infliximab was found as the most associated with GBM (WHO: ROR = 7.41 (5.19–10.57), FAERS: ROR = 2.80 [1.89–4.15]), and adalimumab showed significant correlation (WHO: ROR = 3.54 [2.58–4.89], FAERS: ROR = 1.99 [1.41–2.80]), too. As with the other immunosuppression-related tumors, anti-TNF may in fact reduce the activity of the immune system in inhibiting cancer cells in the brain [86]. It should be noted, however, that RORs derived from pharmacovigilance databases are conceptually different from the fold-increased risk estimates used for other cancer types in IBD patients and cannot be directly compared within a unified risk framework.

Regarding GBM diagnosis, according to the National Cancer Institute, brain tumors are normally diagnosed through brain imaging followed by histopathological evaluation of biopsies. Yet, because of the histological similarity between cancer subtypes, pathologists cannot always diagnose the correct cancer, which leads to higher probability of negative outcomes. Diagnosis through biomarkers would be much less invasive and compliance would most certainly be higher. Prediction or early diagnosis of glioblastoma multiforme (GBM), the most common brain glioma, would increase the life expectancy of patients and, thus, it is of the utmost importance.

For this reason, Tichy and colleagues in 2015 correlated levels of the glial fibrillary acidic protein (GFAP) with histopathological findings (33 patients with GBM and 132 healthy controls). Protein concentration levels in the plasma were assessed by electrochemiluminometric immunoassay for the in vitro quantitative determination of GFAP in human serum and plasma (Elecsys^®^ GFAP prototype test), and they were higher in GBM patients compared to controls. Specifically, a serum titre ≥ 0.01 μg/L was associated with a sensitivity of 85% and a specificity of 70% in detection of GBM from other entities [87].

Mass spectrometry techniques have also been implemented to find serum biomarkers for this specific tumor. Popescu et al. used LC/MS-MS combination to identify biomarkers, and ELISA and Western blot analysis validated their results. Specifically, this research group used SELDI-TOF MS to understand the serum protein expression profile in GBM. This analysis highlighted three putative biomarkers: S100A8, S100A9 and CXCL4 [39].

Both GFAP and S100 calcium-binding proteins (S100A/B) are markers of astrocytic damage. This could mean that they play a role either in the pathogenesis or in the response of glioblastoma, which is in actuality an astrocytoma [88]. However, the understanding of this tumor is still poor and further research is needed.

## 4. Discussion

Inflammatory bowel diseases (IBD) remain only partially understood. In fact, current therapeutic approaches solely focus on inducing and maintaining remission, and they do not address the root cause of the condition, which remains unclear. The increased risk of cancer development is the most serious complication of ulcerative colitis and Crohn’s disease. Therefore, early cancer prevention and careful surveillance of IBD patients are essential.

To achieve this, diagnostic strategies must evolve. The current gold-standard methods, such as endoscopy and imaging techniques can be effective, but they are often invasive, expensive, and not suitable for frequent screening. Moreover, they may fail to detect neoplastic changes at very early stages. There is an urgent need for minimally invasive, highly specific, and sensitive techniques that can identify or predict cancer at its earliest onset, allowing timely and more effective interventions.

One promising approach is the use of protein biomarkers. In this context, proteomics emerges as a crucial tool: not only in enhancing our understanding of IBD pathogenesis but also in identifying and detecting biomarkers for cancer screening in these patients. A notable example is the case of small bowel cancer, where the protein Leucine-rich alpha-2-glycoprotein (LRG) was approved as a cancer biomarker in Japan in 2020 [62]. This success highlights the potential of proteomics as possible future clinical tools. Proteomic techniques used in clinics are also improving: in fact, although ELISA remains the gold standard, mass spectrometry has also started to be implemented in the clinical context, especially, the SELDI-TOF MS analysis. Another interesting approach for implementing proteomics in clinical settings is MRM (Multiple Reaction Monitoring) mass spectrometry. Once candidate biomarkers are discovered (with higher resolution MS techniques, such as Orbitrap), MRM detects specific peptides derived from target proteins. This means that routine patient samples, such as blood, are sufficient for quantifying biomarkers using MRM. The technique allows precise measurement of selected proteins in complex biological matrices. The technique allows precise measurement of selected proteins in complex biological matrices, eliminating the requirement for antibodies. This is particularly advantageous since antibodies are often costly, time-consuming to generate, and not always available for the proteins of interest. Moreover, MRM assays can be multiplexed, enabling the simultaneous analysis of multiple biomarkers in a single run, which increases efficiency and reduces sample requirements. This proteomic approach combines the quantitative accuracy of mass spectrometry with the clinical routines, representing a promising tool for clinical screening of IBD-related cancer markers [89].

Challenges still remain in many fields, one example being the case of glioblastoma: despite the severity of the disease, very few reliable biomarkers are available, and further proteomic research is clearly needed.

Applying proteomic technologies to IBD-related cancer screening could mark a significant turning point. The integration of reliable biomarkers into clinical practice would not only improve patients’ earlier diagnosis but could also serve as a more personalized and effective therapeutic strategy. All the examples of potential biomarkers discussed in the review are summarized in Table 7.

## 5. Conclusions

Proteomic approaches have significantly advanced our understanding of the molecular landscape underlying inflammatory bowel disease (IBD)-associated cancers, enabling the identification of potential biomarkers with diagnostic, prognostic, and predictive value. High-throughput techniques, including mass spectrometry-based proteomics and targeted assays, have revealed protein signatures that differentiate malignant transformation from chronic inflammation, highlighting pathways involved in tumor initiation and progression. Despite these promising findings, the utilization of proteomic discoveries in clinical practice remains limited by several challenges. A primary example of this is variability in sample preparation. In fact, the harmonization of sample processing and analysis is essential to minimize variability and allow reproducibility of experiments proposed by researchers. Moreover, studies with large cohorts are essential for robust validation of potential biomarkers discovered by researchers in smaller exploratory studies. For instance, some of the molecules reported in the review (e.g., PON1) were evaluated in small groups of patients, but they have still not been tested for their ability to detect cancer in vast cohorts. Without rigorous validation, their potential to reliably discriminate between cancer and chronic inflammation in IBD remains uncertain.

Clarifying how biomarker levels change in patients with IBD before the development of cancer is crucial, as this high-risk population requires tailored strategies for surveillance and early detection. Identifying markers that can reliably discriminate between chronic inflammation and tumor initiation would greatly enhance their clinical value, because it would enable timely therapeutic strategies to counter carcinogenesis and thereby improve patient prognosis. Furthermore, the development of clinically adaptable assays, particularly multiplexed and minimally invasive formats such as blood- or stool-based proteomic panels, could transform patient monitoring by enabling longitudinal assessment and earlier intervention. Achieving these goals will require sustained collaboration between proteomics researchers, clinicians, and bioinformaticians to establish robust tools for screening, risk stratification, and therapeutic decision-making.

In conclusion, proteomic research is opening new perspectives for understanding and detecting IBD-associated cancers at stages where clinical intervention can still alter disease trajectory, yet translating these advances into routine practice will require rigorous validation, methodological consistency, and integration with established clinical frameworks. Future investigations should prioritize prospective trials and real-world applications to consolidate proteomic biomarkers as reliable tools for early detection, prognosis, and cancer prevention.

## Figures and Tables

**Table 1:** Stool examination diagnostic test for IBD. Information from World Gastroenterology Organisation Global Guidelines [1].

Stool Examination	
Lactoferrin	Used to exclude intestinal inflammation, it is a negative diagnostic test
Calprotectin	Used to measure the activity of IBD

**Table 2:** Blood examination diagnostic tests for IBD. Information from World Gastroenterology Organisation Global Guidelines [1].

Blood Examination	
Erythrocyte sedimentation rate	Levels correlate with inflammation activity
C-reactive protein	Levels correlate with inflammation activity
Antibody tests (with ELISA)	Mostly for microbial agents that increase probability of IBD

**Table 3:** Incidence of IBD across the world. Data from Molodecky et al. [2].

	UC: Highest Annual Incidence (per 100,000 Person-Years)	CD: Highest Annual Incidence (per 100,000 Person-Years)	Study Period
Europe	24.3	12.7	1930–2008
Asia/Middle East	6.3	5.0	1950–2008
North America	19.2	20.2	1920–2004
Australia	11.2	17.4	1967–2008

**Table 4:** Drugs used for the treatment of IBD. Information extracted from DrugBank (accessed 25 July 2025) [18] and the summary of product characteristics of the listed drugs, available on the FDA web site [19].

Drug Class	Drug Compound	Mechanism of Action	Adverse Effects	Increased Cancer Risk
Amino salicylate	5-Aminosalicylic acid (5-ASA)	Not fully understood. Possible inhibition of COX enzyme.	Nausea, vomiting, abdominal pain, tachypnea, hyperpnea, neurologic symptoms	No
Local corticosteroids	Budesonide	Binding to glucocorticoid receptor inhibits gene expression (i.e., NF-kB, IL-10)	Hypercorticism and adrenal axis suppression	No
Systemic corticosteroids	Prednisolone	Binding to glucocorticoid receptor inhibits gene expression (i.e., NF-kB, IL-10)	Gastrointestinal disturbances, insomnia, and restlessness	Not certain
Immuno- modulators	6-mercaptopurine	Interferes with nucleic acid synthesis by inhibiting purine metabolism	Nausea, vomiting, and diarrhea; myelosuppression, liver dysfunction, gastroenteritis	Risk of developing skin cancer and leukemia under investigation
	Azathioprine (prodrug of 6-mercaptopurine)	Immunosuppressive: modulation of rac1 induces T cell apoptosis	Bone marrow hypoplasia, bleeding, and infection, which may progress to death	Risk of developing skin cancer and lymphoma under investigation
	Methotrexate	Inhibition of cell division by inhibiting nucleotide synthesis	Nausea, vomiting, bone marrow suppression, gastrointestinal ulceration & bleeding	Not certain
TNFα antagonists	Infliximab	Binding to TNFα: disruption of the proinflammatory cascade signaling. Can activate ADCC	Recurrent infections, hepatotoxicity, infusion reactions, allergic reactions	Risk of developing lymphoma and other tumors under investigation
	Adalimumab	Binding to TNFα: disruption of the proinflammatory cascade signaling. Can activate ADCC	Skin rash, swelling, difficulty breathing or swallowing, dyspnea, allergic reactions	Risk of developing leukemia and other tumors under investigation
	Certolizumab	Binding to TNFα: disruption of the proinflammatory cascade signaling. Cannot activate ADCC	Recurrent infections, skin rash, fatigue, hepatotoxicity, allergic reactions	Risk of developing lymphoma and skin cancer under investigation
IL-12 & IL-23 antagonists	Ustekinumab	Binding to IL-12 and IL-23 prevents receptor-mediated responses	Recurrent infections, allergic reactions, itching, diarrhea, nausea, fatigue, bleeding	No
	Risankizumab	Binding to IL-23 inhibits the differentiation of Th17 cells, preventing inflammation	Recurrent infections, headache, itching, fatigue	No
Integrin blockers	Vedolizumab	Binding to the α4β7 integrin prevents homing of T-lymphocytes to gut lymph tissue	Recurrent infections, rash, gastrointestinal symptoms (stypsis, anal abscess, etc.)	No
Janus kinase inhibitors	Upadacitinib	Inhibition of proinflammatory tyrosine-protein kinase JAK1	Infections of upper airways, neutropenia, nausea, cough, hypercholesterolemia	Risk of developing non-melanoma skin cancer under investigation

**Table 5:** Incidence, death rate and 5-year relative survival of different cancer types in the USA, data from the National Cancer Institute of the NIH.

Cancer	Incidence (New Cases of Cancer per 100,000 Men and Women per Year)	Death Rate (Deaths per 100,000 Men and Women per Year)	5-Year Relative Survival (%)
Colorectal cancer	37.1	12.9	65.4
Small bowel cancer	2.6	0.4	71.1
Cholangiocarcinoma	9.4	6.6	22
Melanoma skin cancer	21.9	2.0	94.7
Non-Hodgkin lymphoma	5.6	1.7	64.8
Glioblastoma	6.1	4.4	33
Breast cancer	130.8	19.2	91.7
Lung cancer	47.8	31.5	28.1

**Table 6:** Comparison of main proteomic techniques based on cost, sensitivity, throughput level, diagnostic use, and biomarker discovery potential.

Proteomic Technique	Advantages	Disadvantages	Ref.
ELISA	High specificity; widely used in diagnostics; cost-effective; easy to automate	Limited to known targets; not suitable for biomarker discovery	[30]
LATEX TURBIDIMETRIC ASSAY	Rapid and inexpensive; good for routine diagnostics	Low sensitivity and specificity; limited to known proteins	[38]
Orbitrap MS	High resolution and mass accuracy; excellent for biomarker discovery	High cost; requires expert handling; lower throughput than some other MS types	[31]
MALDI-TOF MS	High-throughput; low cost per sample; rapid analysis	Requires pure samples; less effective for complex mixtures	[31]
ESI-TOF MS	High sensitivity; good for complex mixtures and quantitative analysis	More expensive than MALDI; lower throughput	[31]
SELDI-TOF MS	Suitable for biomarker profiling in diagnostics	Lower resolution and reproducibility; limited to surface-bound proteins	[39]
SWATH-MS	High reproducibility; quantitative; ideal for biomarker discovery; high-throughput	Requires spectral libraries; complex data analysis; high cost	[31]

**Table 7:** Summary of potential biomarkers for different cancer types, their corresponding biological matrices, detection techniques, and current status in clinical diagnostics.

Cancer	Potential Biomarker	Biological Matrix	Technique of Detection	Already Used in Diagnostics	Ref.
Colorectal Cancer	PON1	Blood serum	SWATH-MS	No	[54]
	PRB1 (fragment GG-17)	Feces	Orbitrap MS	No	[58]
Small Bowel Cancer	LRG	Blood serum	Latex turbidimetric immunoassay	Yes	[38]
Cholangiocarcinoma	CA19-9	Blood serum	ELISA	Yes	[65,66]
	CCTγ and S100A9	Liver biopsy	MALDI-TOF	No	[68]
Skin Cancer (nodular BCC subtype)	CYP2W1 and NTRK3	Skin biopsy	Mass Spectrometry	No	[74]
Lymphoma	CXCL13	Blood	ELISA	No	[80]
EBV-related lymphoma	ZEBRA and RTA	Blood	PCR	No	[83]
Glioblastoma	GFAP	Blood serum	ELISA	No	[87]
	S100A8/A9	Blood serum	SELDI-TOF	No	[39]

## Data Availability

No new data were created or analyzed in this study.

## Abbreviations

The following abbreviations are used in this manuscript:

IBD	inflammatory bowel disease
UC	ulcerative colitis
CD	Crohn’s disease
IBD-U	unclassified IBD
GI	gastrointestinal
GWAS	genome-wide association studies
STAT3	Signal Transducer and Activator of Transcription 3
AIEC	Adherent-Invasive Escherichia coli
ELISA	enzyme-linked immunosorbent assay
CRP	C-reactive protein
ECCO	European Crohn’s and Colitis Organisation
5-ASA	5-Aminosalicylic acid
TNFα	tumor necrosis factor α
ADCC	antibody-dependent cell-mediated cytotoxicity
S1P	Sphingosine-1-phosphate
SIR	Standardized Incidence Ratio
CRC	colorectal cancer
SBC	small bowel cancer
SBA	small bowel adenocarcinoma
ALL	acute lymphocytic leukemia
6-TGN	6-thioguanine nucleotides
MMR	mismatch repair
MS	mass spectrometry
TOF	time-of-flight
ESI	electrospray ionization
MALDI	matrix-assisted laser desorption/ionization
SELDI	surface-enhanced laser desorption/ionization
MRM	multiple reaction monitoring
PSC	primary sclerosing cholangitis
CCA	cholangiocarcinoma
NMSC	non-melanoma skin cancer
BCC	basal cell carcinoma
EBV	Epstein–Barr virus

## References

[1] Bernstein C.N., Eliakim A., Fedail S., Fried M., Gearry R., Goh K.-L., Hamid S., Khan A.G., Khalif I., Ng S.C. (2016). World Gastroenterology Organisation Global Guidelines Inflammatory Bowel Disease. J. Clin. Gastroenterol..

[2] Molodecky N.A., Soon I.S., Rabi D.M., Ghali W.A., Ferris M., Chernoff G., Benchimol E.I., Panaccione R., Ghosh S., Barkema H.W. (2012). Increasing Incidence and Prevalence of the Inflammatory Bowel Diseases with Time, Based on Systematic Review. Gastroenterology.

[3] Lophaven S.N., Lynge E., Burisch J. (2017). The Incidence of Inflammatory Bowel Disease in Denmark 1980–2013: A Nationwide Cohort Study. Aliment. Pharmacol. Ther..

[4] Lin D., Jin Y., Shao X., Xu Y., Ma G., Jiang Y., Xu Y., Jiang Y., Hu D. (2024). Global, Regional, and National Burden of Inflammatory Bowel Disease, 1990–2021: Insights from the Global Burden of Disease 2021. Int. J. Color. Dis..

[5] Park J., Jeong G.H., Song M., Yon D.K., Lee S.W., Koyanagi A., Jacob L., Kostev K., Dragioti E., Radua J. (2023). The Global, Regional, and National Burden of Inflammatory Bowel Diseases, 1990–2019: A Systematic Analysis for the Global Burden of Disease Study 2019. Dig. Liver Dis..

[6] Zhang Y.Z., Li Y.Y. (2014). Inflammatory Bowel Disease: Pathogenesis. World J. Gastroenterol..

[7] Jostins L., Ripke S., Weersma R.K., Duerr R.H., McGovern D.P., Hui K.Y., Lee J.C., Schumm L.P., Sharma Y., Anderson C.A. (2012). Host-Microbe Interactions Have Shaped the Genetic Architecture of Inflammatory Bowel Disease. Nature.

[8] Kaplan G.G., Hubbard J., Korzenik J., Sands B.E., Panaccione R., Ghosh S., Wheeler A.J., Villeneuve P.J. (2010). The Inflammatory Bowel Diseases and Ambient Air Pollution: A Novel Association. Am. J. Gastroenterol..

[9] Darfeuille-Michaud A., Boudeau J., Bulois P., Neut C., Glasser A.L., Barnich N., Bringer M.A., Swidsinski A., Beaugerie L., Colombel J.F. (2004). High Prevalence of Adherent-Invasive Escherichia Coli Associated with Ileal Mucosa in Crohn’s Disease. Gastroenterology.

[10] Putignani L., Oliva S., Isoldi S., Del Chierico F., Carissimi C., Laudadio I., Cucchiara S., Stronati L. (2021). Fecal and Mucosal Microbiota Profiling in Pediatric Inflammatory Bowel Diseases. Eur. J. Gastroenterol. Hepatol..

[11] Zhou L., Ivanov I.I., Spolski R., Min R., Shenderov K., Egawa T., Levy D.E., Leonard W.J., Littman D.R. (2007). IL-6 Programs TH-17 Cell Differentiation by Promoting Sequential Engagement of the IL-21 and IL-23 Pathways. Nat. Immunol..

[12] Gu C., Wu L., Li X. (2013). IL-17 Family: Cytokines, Receptors and Signaling. Cytokine.

[13] Kotla N.G., Rochev Y. (2023). IBD Disease-Modifying Therapies: Insights from Emerging Therapeutics. Trends Mol. Med..

[14] Harbord M., Eliakim R., Bettenworth D., Karmiris K., Katsanos K., Kopylov U., Kucharzik T., Molnár T., Raine T., Sebastian S. (2017). Third European Evidence-Based Consensus on Diagnosis and Management of Ulcerative Colitis. Part 2: Current Management. J. Crohns Colitis.

[15] Raine T., Bonovas S., Burisch J., Kucharzik T., Adamina M., Annese V., Bachmann O., Bettenworth D., Chaparro M., Czuber-Dochan W. (2022). ECCO Guidelines on Therapeutics in Ulcerative Colitis: Medical Treatment. J. Crohns Colitis.

[16] Torres J., Bonovas S., Doherty G., Kucharzik T., Gisbert J.P., Raine T., Adamina M., Armuzzi A., Bachmann O., Bager P. (2020). ECCO Guidelines on Therapeutics in Crohn’s Disease: Medical Treatment. J. Crohns Colitis.

[17] Gordon H., Minozzi S., Kopylov U., Verstockt B., Chaparro M., Buskens C., Warusavitarne J., Agrawal M., Allocca M., Atreya R. (2024). ECCO Guidelines on Therapeutics in Crohn’s Disease: Medical Treatment. J. Crohns Colitis.

[18] Knox C., Wilson M., Klinger C.M., Franklin M., Oler E., Wilson A., Pon A., Cox J., Chin N.E., Strawbridge S.A. (2024). DrugBank 6.0: The DrugBank Knowledgebase for 2024. Nucleic Acids Res..

[19] Craigle V. (2007). MedWatch: The FDA Safety Information and Adverse Event Reporting Program. J. Med. Libr. Assoc..

[20] Noor N.M., Bourke A., Subramanian S. (2024). Review Article: Novel Therapies in Inflammatory Bowel Disease—An Update for Clinicians. Aliment. Pharmacol. Ther..

[21] Bargen J.A. (1928). Chronic Ulcerative Colitis Associated with Malignant Disease. Arch. Surg..

[22] Ekbom A. (1998). Clinical Review Risk Factors and Distinguishing Features of Cancer in IBD. Inflamm. Bowel Dis..

[23] Scharl S., Barthel C., Rossel J.B., Biedermann L., Misselwitz B., Schoepfer A.M., Straumann A., Vavricka S.R., Rogler G., Scharl M. (2019). Malignancies in Inflammatory Bowel Disease: Frequency, Incidence and Risk Factors—Results from the Swiss IBD Cohort Study. Am. J. Gastroenterol..

[24] Biancone L., Armuzzi A., Scribano M.L., Castiglione F., D’Incà R., Orlando A., Papi C., Daperno M., Vecchi M., Riegler G. (2020). Cancer Risk in Inflammatory Bowel Disease: A 6-Year Prospective Multicenter Nested Case-Control IG-IBD Study. Inflamm. Bowel Dis..

[25] Fotoohi A.K., Coulthard S.A., Albertioni F. (2010). Thiopurines: Factors Influencing Toxicity and Response. Biochem. Pharmacol..

[26] Ledder O. (2022). The Question That Doesn’t Seem to Go Away: Cancer Risk of Anti-TNF Therapy. Dig. Dis. Sci..

[27] Wu S., Xie S., Yuan C., Yang Z., Liu S., Zhang Q., Sun F., Wu J., Zhan S., Zhu S. (2023). Inflammatory Bowel Disease and Long-Term Risk of Cancer: A Prospective Cohort Study among Half a Million Adults in UK Biobank. Inflamm. Bowel Dis..

[28] Tyers M., Mann M. (2003). From Genomics to Proteomics. Nature.

[29] Maes E., Mertens I., Valkenborg D., Pauwels P., Rolfo C., Baggerman G. (2015). Proteomics in Cancer Research: Are We Ready for Clinical Practice?. Crit. Rev. Oncol. Hematol..

[30] Ahsan H. (2022). Monoplex and Multiplex Immunoassays: Approval, Advancements, and Alternatives. Comp. Clin. Path.

[31] Shuken S.R. (2023). An Introduction to Mass Spectrometry-Based Proteomics. J. Proteome Res..

[32] Duarte T.T., Spencer C.T. (2016). Personalized Proteomics: The Future of Precision Medicine. Proteomes.

[33] Frantzi M., Metzger J., Banks R.E., Husi H., Klein J., Dakna M., Mullen W., Cartledge J.J., Schanstra J.P., Brand K. (2014). Discovery and Validation of Urinary Biomarkers for Detection of Renal Cell Carcinoma. J. Proteom..

[34] Frantzi M., Van Kessel K.E., Zwarthoff E.C., Marquez M., Rava M., Malats N., Merseburger A.S., Katafigiotis I., Stravodimos K., Mullen W. (2016). Development and Validation of Urine-Based Peptide Biomarker Panels for Detecting Bladder Cancer in a Multi-Center Study. Clin. Cancer Res..

[35] Njoku K., Pierce A., Geary B., Campbell A.E., Kelsall J., Reed R., Armit A., Da Sylva R., Zhang L., Agnew H. (2023). Quantitative SWATH-Based Proteomic Profiling of Urine for the Identification of Endometrial Cancer Biomarkers in Symptomatic Women. Br. J. Cancer.

[36] Xu D., Zhu X., Ren J., Huang S., Xiao Z., Jiang H., Tan Y. (2022). Quantitative Proteomic Analysis of Cervical Cancer Based on TMT-Labeled Quantitative Proteomics. J. Proteom..

[37] Whiteaker J.R., Lin C., Kennedy J., Hou L., Trute M., Sokal I., Yan P., Schoenherr R.M., Zhao L., Voytovich U.J. (2011). A Targeted Proteomics-Based Pipeline for Verification of Biomarkers in Plasma. Nat. Biotechnol..

[38] Asonuma K., Kobayashi T., Kikkawa N., Nakano M., Sagami S., Morikubo H., Miyatani Y., Hojo A., Fukuda T., Hibi T. (2023). Optimal Use of Serum Leucine-Rich Alpha-2 Glycoprotein as a Biomarker for Small Bowel Lesions of Crohn’s Disease. Inflamm. Intest. Dis..

[39] Popescu I.D., Codrici E., Albulescu L., Mihai S., Enciu A.M., Albulescu R., Tanase C.P. (2014). Potential Serum Biomarkers for Glioblastoma Diagnostic Assessed by Proteomic Approaches. Proteome Sci..

[40] Salomon B., Sudhakar P., Bergemalm D., Andersson E., Grännö O., Carlson M., Hedin C.R.H., Söderholm J.D., Öhman L., Ungaro R.C. (2024). Characterization of Inflammatory Bowel Disease Heterogeneity Using Serum Proteomics: A Multicenter Study. J. Crohns Colitis.

[41] Basso D., Padoan A., D’Incà R., Arrigoni G., Scapellato M.L., Contran N., Franchin C., Lorenzon G., Mescoli C., Moz S. (2020). Peptidomic and Proteomic Analysis of Stool for Diagnosing IBD and Deciphering Disease Pathogenesis. Clin. Chem. Lab. Med..

[42] Park J.M., Han N.Y., Han Y.M., Chung M.K., Lee H.K., Ko K.H., Kim E.H., Hahm K.B. (2014). Predictive Proteomic Biomarkers for Inflammatory Bowel Disease-Associated Cancer: Where Are We Now in the Era of the next Generation Proteomics?. World J. Gastroenterol..

[43] Angriman I., Scarpa M., D’Incà R., Basso D., Ruffolo C., Polese L., Sturniolo G.C., D’Amico D.F., Plebani M. (2007). Enzymes in Feces: Useful Markers of Chronic Inflammatory Bowel Disease. Clin. Chim. Acta.

[44] Vaiopoulou A., Gazouli M., Theodoropoulos G., Zografos G. (2012). Current Advantages in the Application of Proteomics in Inflammatory Bowel Disease. Dig. Dis. Sci..

[45] Tatsumi N., Kushima R., Vieth M., Mukaisho K.I., Kakinoki R., Okabe H., Borchard F., Stolte M., Okanoue T., Hattori T. (2006). Cytokeratin 7/20 and Mucin Core Protein Expression in Ulcerative Colitis-Associated Colorectal Neoplasms. Virchows Arch..

[46] Hashimoto K., Saigusa S., Araki T., Tanaka K., Okita Y., Fujikawa H., Kawamura M., Okugawa Y., Toiyama Y., Inoue Y. (2013). Correlation of CCL20 Expression in Rectal Mucosa with the Development of Ulcerative Colitis-Associated Neoplasia. Oncol. Lett..

[47] Yeo M., Kim D.K., Park H.J., Oh T.Y., Kim J.H., Cho S.W., Paik Y.K., Nahm K.B. (2006). Loss of Transgelin in Repeated Bouts of Ulcerative Colitis-Induced Colon Carcinogenesis. Proteomics.

[48] May D., Pan S., Crispin D.A., Lai K., Bronner M.P., Hogan J., Hockenbery D.M., McIntosh M., Brentnall T.A., Chen R. (2011). Investigating Neoplastic Progression of Ulcerative Colitis with Label-Free Comparative Proteomics. J. Proteome Res..

[49] Murata M. (2018). Inflammation and Cancer. Environ. Health Prev. Med..

[50] Jewel Samadder N., Valentine J.F., Guthery S., Singh H., Bernstein C.N., Wan Y., Wong J., Boucher K., Pappas L., Rowe K. (2017). Colorectal Cancer in Inflammatory Bowel Diseases: A Population-Based Study in Utah. Dig. Dis. Sci..

[51] Eaden J.A., Abrams K.R., Mayberry J.F. (2001). The Risk of Colorectal Cancer in Ulcerative Colitis: A Meta-Analysis. Gut.

[52] Peyrin-Biroulet L., Lepage C., Jooste V., Guéant J.L., Faivre J., Bouvier A.M. (2012). Colorectal Cancer in Inflammatory Bowel Diseases: A Population-Based Study (1976–2008). Inflamm. Bowel Dis..

[53] Selinger C.P., Andrews J.M., Titman A., Norton I., Jones D.B., McDonald C., Barr G., Selby W., Leong R., Andrews J. (2014). Long-Term Follow-up Reveals Low Incidence of Colorectal Cancer, but Frequent Need for Resection, Among Australian Patients with Inflammatory Bowel Disease. Clin. Gastroenterol. Hepatol..

[54] Ahn S.B., Sharma S., Mohamedali A., Mahboob S., Redmond W.J., Pascovici D., Wu J.X., Zaw T., Adhikari S., Vaibhav V. (2019). Potential Early Clinical Stage Colorectal Cancer Diagnosis Using a Proteomics Blood Test Panel. Clin. Proteom..

[55] Boehm D., Krzystek-Korpacka M., Neubauer K., Matusiewicz M., Berdowska I., Zielinski B., Paradowski L., Gamian A. (2009). Paraoxonase-1 Status in Crohn’s Disease and Ulcerative Colitis. Inflamm. Bowel Dis..

[56] Devarajan A., Shih D., Reddy S.T. (2014). Inflammation, Infection, Cancer 5 and All That…the Role of Paraoxonases. Adv. Exp. Med. Biol..

[57] Tratenšek A., Locatelli I., Grabnar I., Drobne D., Vovk T. (2024). Oxidative Stress-Related Biomarkers as Promising Indicators of Inflammatory Bowel Disease Activity: A Systematic Review and Meta-Analysis. Redox Biol..

[58] Contran N., Arrigoni G., Battisti I., D’Incà R., Angriman I., Franchin C., Scapellato M.L., Padoan A., Moz S., Aita A. (2024). Colorectal Cancer and Inflammatory Bowel Diseases Share Common Salivary Proteomic Pathways. Sci. Rep..

[59] Chan M., Bennick A. (2001). Proteolytic Processing of a Human Salivary Proline-Rich Protein Precursor by Proprotein Convertases. Eur. J. Biochem..

[60] Bojesen R.D., Riis L.B., Høgdall E., Nielsen O.H., Jess T. (2017). Inflammatory Bowel Disease and Small Bowel Cancer Risk, Clinical Characteristics, and Histopathology: A Population-Based Study. Clin. Gastroenterol. Hepatol..

[61] Lin M., Liu J., Zhang F., Qi G., Tao S., Fan W., Chen M., Ding K., Zhou F. (2022). The Role of Leucine-Rich Alpha-2-Glycoprotein-1 in Proliferation, Migration, and Invasion of Tumors. J. Cancer Res. Clin. Oncol..

[62] Shimoyama T., Yamamoto T., Yoshiyama S., Nishikawa R., Umegae S. (2023). Leucine-Rich Alpha-2 Glycoprotein Is a Reliable Serum Biomarker for Evaluating Clinical and Endoscopic Disease Activity in Inflammatory Bowel Disease. Inflamm. Bowel Dis..

[63] Huai J.P., Ding J., Ye X.H., Chen Y.P. (2014). Inflammatory Bowel Disease and Risk of Cholangiocarcinoma: Evidence from a Meta-Analysis of Population-Based Studies. Asian Pac. J. Cancer Prev..

[64] Yu J., Refsum E., Helsingen L.M., Folseraas T., Ploner A., Wieszczy P., Barua I., Jodal H.C., Melum E., Løberg M. (2022). Risk of Hepato-Pancreato-Biliary Cancer Is Increased by Primary Sclerosing Cholangitis in Patients with Inflammatory Bowel Disease: A Population-Based Cohort Study. United Eur. Gastroenterol. J..

[65] Liang B., Zhong L., He Q., Wang S., Pan Z., Wang T., Zhao Y. (2015). Diagnostic Accuracy of Serum CA19-9 in Patients with Cholangiocarcinoma: A Systematic Review and Meta-Analysis. Med. Sci. Monit..

[66] Shirazi K.M., Hosseinzadeh Y., Nourpanah Z., Shirazinezhad A.M., Nikniaz Z. (2020). The Value of Serum CA19-9 in Predicting Primary Sclerosing Cholangitis in Patients with Ulcerative Colitis. Adv. Dig. Med..

[67] Luo G., Jin K., Deng S., Cheng H., Fan Z., Gong Y., Qian Y., Huang Q., Ni Q., Liu C. (2021). Roles of CA19-9 in Pancreatic Cancer: Biomarker, Predictor and Promoter. Biochim. Biophys Acta Rev Cancer.

[68] Shi Y., Deng X., Zhan Q., Shen B., Jin X., Zhu Z., Chen H., Li H., Peng C. (2013). A Prospective Proteomic-Based Study for Identifying Potential Biomarkers for the Diagnosis of Cholangiocarcinoma. J. Gastrointest. Surg..

[69] Ma J., Grant C.E., Plagens R.N., Barrett L.N., Guisbert K.S.K., Guisbert E. (2017). Cellular Proteomes Drive Tissue-Specific Regulation of the Heat Shock Response. G3 Genes. Genomes Genet..

[70] Wang S., Song R., Wang Z., Jing Z., Wang S., Ma J. (2018). S100A8/A9 in Inflammation. Front Immunol.

[71] Long M.D., Martin C.F., Pipkin C.A., Herfarth H.H., Sandler R.S., Kappelman M.D. (2012). Risk of Melanoma and Nonmelanoma Skin Cancer among Patients with Inflammatory Bowel Disease. Gastroenterology.

[72] Huang S.Z., Liu Z.C., Liao W.X., Wei J.X., Huang X.W., Yang C., Xia Y.H., Li L., Ye C., Dai S.X. (2019). Risk of Skin Cancers in Thiopurines-Treated and Thiopurines-Untreated Patients with Inflammatory Bowel Disease: A Systematic Review and Meta-Analysis. J. Gastroenterol. Hepatol..

[73] Osterman M.T., Sandborn W.J., Colombel J.F., Robinson A.M., Lau W., Huang B., Pollack P.F., Thakkar R.B., Lewis J.D. (2014). Increased Risk of Malignancy with Adalimumab Combination Therapy, Compared with Monotherapy, for Crohn’s Disease. Gastroenterology.

[74] Berl A., Shir-Az O., Genish I., Biran H., Mann D., Singh A., Wise J., Kravtsov V., Kidron D., Golberg A. (2023). Exploring Multisite Heterogeneity of Human Basal Cell Carcinoma Proteome and Transcriptome. PLoS ONE.

[75] Zhao Y., Wan D., Yang J., Hammock B.D., De Montellano P.R.O. (2016). Catalytic Activities of Tumor-Specific Human Cytochrome P450 CYP2W1 toward Endogenous Substrates. Drug Metab. Dispos..

[76] Wood L.D., Calhoun E.S., Silliman N., Ptak J., Szabo S., Powell S.M., Riggins G.J., Wang T.L., Yan H., Gazdar A. (2006). Somatic Mutations of GUCY2F, EPHA3, and NTRK3 in Human Cancers. Hum. Mutat..

[77] Beaugerie L., Brousse N., Marie Bouvier A., Frédéric Colombel J., Lémann M., Cosnes J., Hébuterne X., Cortot A., Bouhnik Y., Pierre Gendre J. (2009). Lymphoproliferative Disorders in Patients Receiving Thiopurines for Infl Ammatory Bowel Disease: A Prospective Observational Cohort Study. Lancet.

[78] Khan N., Abbas A.M., Lichtenstein G.R., Loftus E.V., Bazzano L.A. (2013). Risk of Lymphoma in Patients with Ulcerative Colitis Treated with Thiopurines: A Nationwide Retrospective Cohort Study. Gastroenterology.

[79] Vos A.C.W., Bakkal N., Minnee R.C., Casparie M.K., De Jong D.J., Dijkstra G., Stokkers P., Van Bodegraven A.A., Pierik M., Van Der Woude C.J. (2011). Risk of Malignant Lymphoma in Patients with Inflammatory Bowel Diseases: A Dutch Nationwide Study. Inflamm. Bowel Dis..

[80] Marchesi F., Martin A.P., Thirunarayanan N., Devany E., Mayer L., Grisotto M.G., Furtado G.C., Lira S.A. (2009). CXCL13 Expression in the Gut Promotes Accumulation of IL-22-Producing Lymphoid Tissue-Inducer Cells, and Formation of Isolated Lymphoid Follicles. Mucosal Immunol..

[81] Spåth F., Wibom C., Krop E.J.M., Johansson A.S., Bergdahl I.A., Vermeulen R., Melin B. (2017). Biomarker Dynamics in B-Cell Lymphoma: A Longitudinal Prospective Study of Plasma Samples up to 25 Years before Diagnosis. Cancer Res..

[82] Wang B., Wang M., Ao D., Wei X. (2022). CXCL13-CXCR5 Axis: Regulation in Inflammatory Diseases and Cancer. Biochim. Biophys. Acta Rev. Cancer.

[83] Lapsia S., Koganti S., Spadaro S., Rajapakse R., Chawla A., Bhaduri-Mcintosh S. (2016). Anti-TNFα Therapy for Inflammatory Bowel Diseases Is Associated with Epstein-Barr Virus Lytic Activation. J. Med. Virol..

[84] De Palma G., Collins S.M., Bercik P., Verdu E.F. (2014). The Microbiota-Gut-Brain Axis in Gastrointestinal Disorders: Stressed Bugs, Stressed Brain or Both?. J. Physiol..

[85] Ishaq H.M., Yasin R., Mohammad I.S., Fan Y., Li H., Shahzad M., Xu J. (2024). The Gut-Brain-Axis: A Positive Relationship between Gut Microbial Dysbiosis and Glioblastoma Brain Tumour. Heliyon.

[86] Guo M., Luo H., Samii A., Etminan M. (2016). The Risk of Glioblastoma with TNF Inhibitors. Pharmacotherapy.

[87] Tichy J., Spechtmeyer S., Mittelbronn M., Hattingen E., Rieger J., Senft C., Foerch C. (2015). Prospective Evaluation of Serum Glial Fibrillary Acidic Protein (GFAP) as a Diagnostic Marker for Glioblastoma. J. Neurooncol..

[88] Kimura A., Takemura M., Yamamoto Y., Hayashi Y., Saito K., Shimohata T. (2019). Cytokines and Biological Markers in Autoimmune GFAP Astrocytopathy: The Potential Role for Pathogenesis and Therapeutic Implications. J. Neuroimmunol..

[89] Jun J., Gim J., Kim Y., Kim H., Yu S.J., Yeo I., Park J., Yoo J.J., Cho Y.Y., Lee D.H. (2018). Analysis of Significant Protein Abundance from Multiple Reaction-Monitoring Data. BMC Syst. Biol..

